# Seed Total Protein Profiling in Discrimination of Closely Related Pines: Evidence from the *Pinus mugo* Complex

**DOI:** 10.3390/plants9070872

**Published:** 2020-07-09

**Authors:** Konrad Celiński, Joanna Sokołowska, Agata Zemleduch-Barylska, Roman Kuna, Hanna Kijak, Aleksandra Maria Staszak, Aleksandra Wojnicka-Półtorak, Ewa Chudzińska

**Affiliations:** 1 Department of Genetics, Institute of Experimental Biology, Faculty of Biology, School of Natural Sciences, Adam Mickiewicz University, Uniwersytetu Poznańskiego 6, 61-614 Poznań, Poland; joasok@amu.edu.pl (J.S.); olawp@amu.edu.pl (A.W.-P.); evpell@amu.edu.pl (E.C.); 2 Department of Biochemistry and Food Analysis, Faculty of Food and Nutrition Sciences, University of Life Sciences, Mazowiecka 48, 60-623 Poznań, Poland; agata.zemleduch@gmail.com; 3 Department of Botany and Genetics, Faculty of Natural Sciences, Constantine the Philosopher University in Nitra, Tr. A. Hlinku 1, 949 74 Nitra, Slovakia; rkuna@ukf.sk; 4 Institute of Dendrology, Polish Academy of Sciences, Parkowa 5, 62-035 Kórnik, Poland; hkijak@man.poznan.pl; 5 Laboratory of Plant Physiology, Department of Plant Biology and Ecology, Faculty of Biology, University of Bialystok, Ciołkowskiego 1J, 15-245 Białystok, Poland; a.staszak@uwb.edu.pl

**Keywords:** seed total proteins, taxonomic discrimination, SDS-PAGE protein profiling, species complex, Pinaceae

## Abstract

The *Pinus mugo* complex includes several dozen closely related European mountain pines. The discrimination of specific taxa within this complex is still extremely challenging, although numerous methodologies have been used to solve this problem, including morphological and anatomical analyses, cytological studies, allozyme variability, and DNA barcoding, etc. In this study, we used the seed total protein (STP) patterns to search for taxonomically interesting differences among three closely-related pine taxa from the *Pinus mugo* complex and five more distant species from the Pinaceae family. It was postulated that STP profiling can serve as the backup methodology for modern taxonomic research, in which more sophisticated analyses, i.e., based on the DNA barcoding approach, have been found to be useless. A quantitative analysis of the STP profiles revealed characteristic electrophoretic patterns for all the analyzed taxa from Pinaceae. STP profiling enabled the discrimination of closely-related pine taxa, even of those previously indistinguishable by chloroplast DNA barcodes. The results obtained in this study indicate that STP profiling can be very useful for solving complex taxonomic puzzles.

## 1. Introduction

In all biological research, reliable and unambiguous identification of the analyzed material is fundamental, as inaccuracies in this regard lead to incorrect and misleading conclusions [1]. The complexity of a biological object, founded by both genetic and environmental determinants, makes it challenging to unambiguously define its phylogenetic relationships and taxonomy. Classic approaches are based on the characteristics of the morphological and anatomical features. However, such taxonomy is severely disturbed by the impact of environmental factors that do not reflect the actual discrimination of the species. In contrast, molecular studies, addressing the genetic determinants with high precision, meet the requirements of unambiguous species discrimination and identification. Moreover, a molecular approach enables insight into the various phenomena and evolutionary processes occurring between species, or the determination of their genetic origin. 

The use of molecular markers in taxonomy and phylogenetics dates back to the 1960s, when protein markers (mainly seed storage proteins) were used in plant taxonomic research [2]. Consequently, the discovery of allozymes variability and their analysis through gel assays enabled insight into genetic diversity and the genetic structure of many different populations [3,4]. Alongside this, the other molecular compounds entered taxonomical studies, including small-molecular weight metabolites (such as those contained in essential oils) as new taxonomical descriptors, leading to the rapid development of chemotaxonomy [5,6,7,8]. Other recent studies have shown that the current taxonomic problems can be successfully addressed using protein pattern analysis for distinguishing *Nepenthes* L. species [9].

Around the 1980s, the spectrum of tools used in taxonomic and phylogenetic research began to expand rapidly, mainly because of the development of various types of DNA markers [10]. Recently, a very interesting concept of DNA barcoding emerged [11,12], relying on the use of one or several highly polymorphic DNA regions for the simple and unambiguous identification and discrimination of species [13,14,15]. Currently, the DNA barcoding approach is being replaced by a comparative analysis of the whole genomes enabled by the advanced sequencing techniques and bioinformatic tools [16]. The so called “super-barcoding” operating at whole genomes was successfully applied to discriminate several taxa [17,18]. Still, as evidenced in the previous reports [1], the use of DNA barcoding does not guarantee a solution to all taxonomic problems. This particularly applies to the discrimination of individual taxa in a group of closely related organisms (species complex). Hence, backup methodologies addressing the other molecular parameters are still welcomed. Moreover, all of the above mentioned methodologies differ in terms of the species discrimination rate (resolution of the given method), level of difficulty, availability and required quantity of the material for the research, or the cost of the analysis. 

Seed total protein (STP) profiling has been used in taxonomic and evolutionary studies since around the 1960s [2]. Using this technique, it has been possible to determine the level of genetic diversity and to estimate thephylogenetic relationships of many species and genera, including *Opuntia* Mill. [19], *Crotalaria* L. [20], *Capsium* L. [21], *Bauhinia* L. [22], *Lathyrus* L. [23], *Consolida* Gray [24], or *Hordeum* L. [25], demonstrating its usefulness in this regard. However, this method has not been widely used in the studies of gymnosperms, including the members of the Pinaceae family. Therefore, little is known about its usefulness in the discrimination of pine taxa.

*Pinus mugo* complex refers to an aggregate of European mountain pines, covering several dozens of different taxa. The most known and the best studied are *Pinus mugo* Turra, *Pinus uncinata* Ramond, and *Pinus uliginosa* Neumann ex Wimmer. The taxonomic and evolutionary status in this complex is still not resolved, which is founded by the following: common usage of synonymous names describing probably the same taxa, sympatric occurrence of the taxa accompanied by the presence of hybrid individuals, and ongoing hybridization processes [26,27,28], which altogether led to difficulties in the discrimination of species with a similar morphology, or inaccurate assigning of individuals to a particular taxa. Taxonomic and evolutionary relationships among closely related taxa in the *Pinus mugo* complex have been studied in detail for many years using various tools and methods, i.e., serological [29], allozymatic [30,31], RAPD markers [32], molecular cytogenetics, and flow cytometry [33,34], as well as the DNA barcoding approach [1]. The obtained results show a similar genetic background and a lack of distinct genetic differences among them. The origins, reciprocal relationships, species discrimination, and the rank of individual taxa in the *Pinus mugo* complex remain puzzling and require further research in order to reach the consensus. On the other hand, recent chemotaxonomic studies on some representatives of the *Pinus mugo* complex revealed species-specific differences in the composition of the essential oils and volatile compounds extracted from needles [35,36], suggesting the need for further research using alternative approaches. 

The present research was undertaken to examine the pattern of STPs of selected taxa from the *Pinus mugo* complex, and to determine the potential usefulness of the STPs profiling method in the discrimination of closely related pine taxa, which has not been previously addressed. The specific goals of this work comprise the following: (1) STP profiling in selected members of the Pinaceae family, including closely related *Pinus mugo*, *Pinus uliginosa,* and *Pinus* × *rhaetica* Brügger from the *Pinus mugo* complex; (2) identification of diagnostic bands, useful in the discrimination of the taxa; (3) conducting cluster analysis and phylogenetic inference based on STP patterns; and finally (4) conclusions on whether the STP-based results obtained here confirm, complement, or contradict our previously obtained data on DNA barcoding and chemotaxonomy in these taxa.

## 2. Results and Discussion

### 2.1. STPs Profiling—Interspecific Comparative Analysis

**Figure 1:**
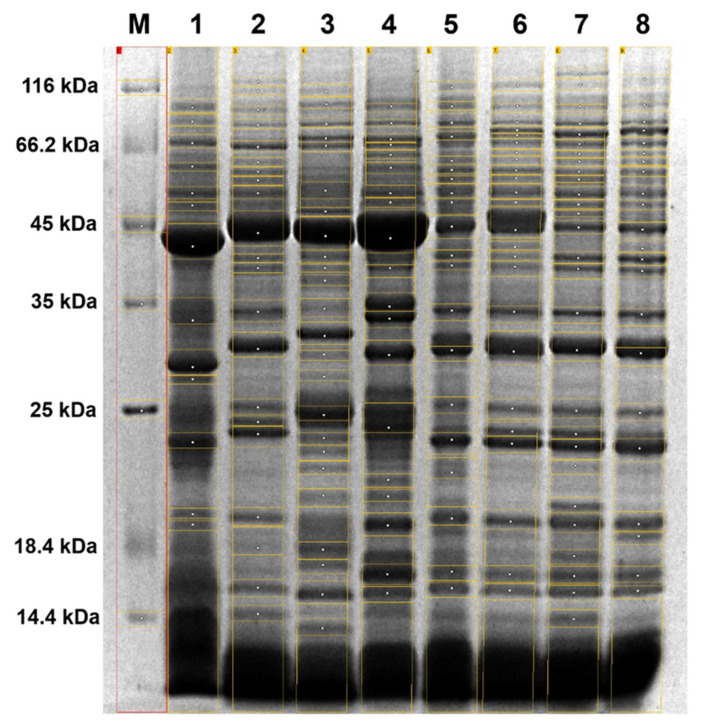
Representative SDS-PAGE image showing seed total protein (STP) profiles from the eight taxa under study. Lane 1: *Abies koreana*, 2: *Pinus nigra* Arn., 3: *Pinus strobus*, 4: *Pseudotsuga menziesii* (Mirbell) Franco, 5: *Pinus sylvestris*, 6: *Pinus* × *rhaetica*, 7: *Pinus uliginosa*, 8: *Pinus mugo*, M: Unstained Protein Molecular Weight Marker (14.4 kDa–116 kDa; Thermo Fisher Scientific, Inc.).

A quantitative analysis of the STP profiles from the eight taxa under study revealed interspecific variations in terms of the band number and their relative mobility value (Rf; Figure 1). 

The macroscopically observed variation between the STP profiles was confirmed using digital image processing tools. The number of bands for individual STPs varied from 13 for *Abies koreana* E.H. Wilson to 28 for *Pinus uliginosa* (Table 1), with the average number of 21.375 bands per separated STP preparation. In total, 66 bands with a different relative mobility value (Rf) were identified, ranging from Rf of 0.04 to 0.87, which correspond to molecular weights from 14.3 kDa to 130.8 kDa.

Surprisingly, only one band out of those 66 (assigned a specific Rf value = 0.22) was present in all of the analyzed STP profiles. Unique bands were identified for seven of the eight analyzed taxa (87.5%), and their numbers ranged from two bands for *Abies koreana*, *Pinus sylvestris* L., and *Pinus mugo*, to seven bands identified for *Pinus strobus* L. Overall, the STP profiles of the *Pinus* taxa were more complex, represented by more bands than the STP profiles of the other analyzed taxa from the Pinaceae family.

As postulated previously, common bands in the protein profile may indicate a hybrid origin of taxa. The usefulness of STP intermediate (hybrid) profiles as a proof of a hybrid origin of species was demonstrated in studies on *Amaranthus* L. [37]. In the present study, one taxon (*Pinus* × *rhaetica*) did not have any species specific protein band, despite the presence of 24 bands in the protein profile, which is a relatively high number. In the scientific literature, *Pinus* × *rhaetica* is sometimes used as a synonymous name of *Pinus uliginosa*, suggesting the existence of only one taxon [27]. In other reports, *Pinus* × *rhaetica* and *Pinus uliginosa* are considered to be hybrid individuals, representing two separate taxa [34]. Even more puzzling, the postulated parental species for the two above-mentioned taxa are exactly the same, i.e., monocormic (*Pinus sylvestris*) and polycormic (*Pinus mugo*) [27].

Previously conducted research clearly indicates the possibility of crossings between taxa from the *Pinus mugo* complex with *Pinus sylvestris*, and the formation of hybrid individuals [28,38,39,40]. The absence of species specific bands in the *Pinus* × *rhaetica* STP profile may not necessarily be evidence of its hybrid origin and intermediate profile (a hybrid of parental taxa profiles). It may equally well indicate that it is a young taxon that has not yet accumulated the relevant mutations, as observable in the STP pattern. However, the higher number of common bands of *Pinus* × *rhaetica* with *Pinus sylvestris* STP profiles (15), than between *Pinus* × *rhaetica* and *Pinus mugo* (13), suggests a closer phylogenetic relationship with the former. Interestingly, *Pinus uliginosa* STPs profile shares 10 and 16 common bands with its putative parents, i.e., *Pinus sylvestris* and *Pinus mugo*, respectively, indicating closer phylogenetic relationship with *Pinus mugo* than with *Pinus sylvestris*. This is the opposite to the second postulated hybrid descendant of the two species—*Pinus* × *rhaetica*. 

**Figure 2:**
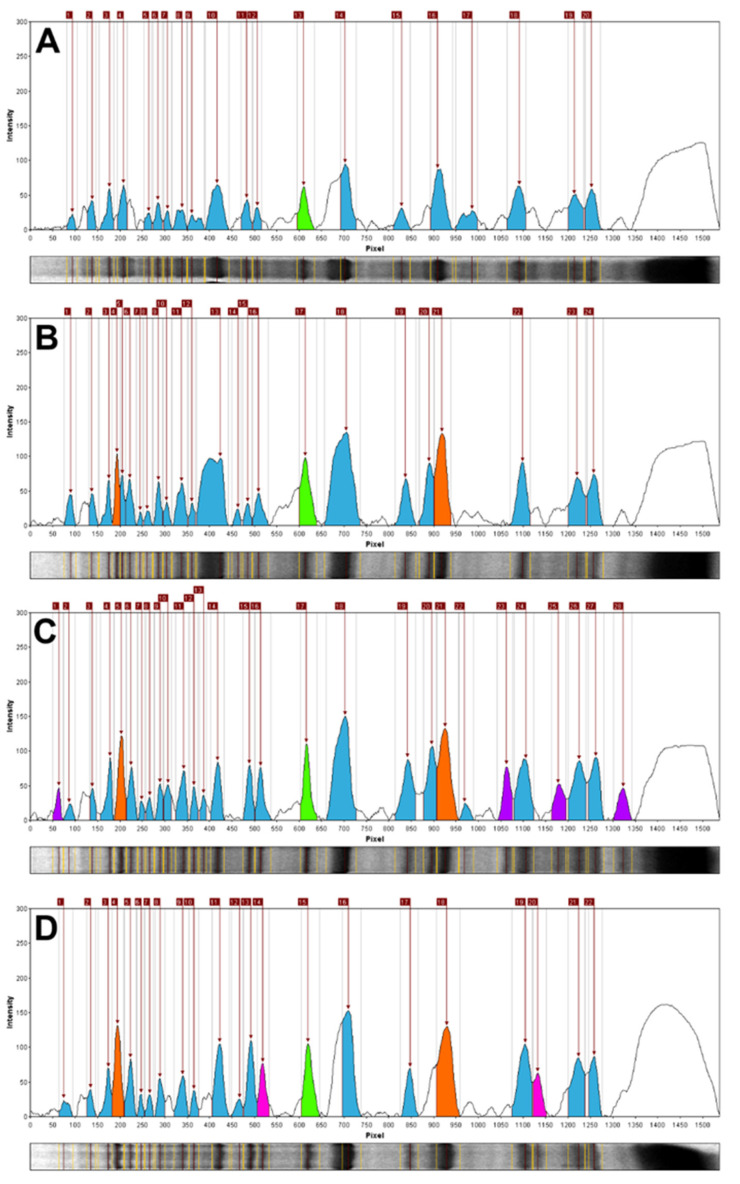
Relative line image plots (densitograms) based on the STP profiling. (**A**): *Pinus* sylvestris, (**B**): *Pinus* × *rhaetica*, (**C**): *Pinus uliginosa,* and (**D**): *Pinus mugo.* Color code: orange—specific and semi specific bands for the *Pinus mugo* complex members (Rf = 0.13 and Rf = 0.60); green—a band specific to the *Pinus* section (Rf = 0.40; purple—diagnostic bands for *Pinus uliginosa* (Rf = 0.04, 0.69, 0.77, and 0.86); magenta—bands specific for *Pinus mugo* (Rf = 0.34 and 0.74); blue—represents the other bands identified in the given profile.

Notably, the densitometric analysis of the STP profiles of *Pinus* × *rhaetica* and *Pinus uliginosa* showed their clear dissimilarity. Both taxa differed in terms of the total number of bands (24 and 28, respectively), as well as in their relative mobility range (Rf of 0.06–0.82 for *Pinus* × *rhaetica* and 0.04–0.86 for *Pinus uliginosa*). Correspondingly, the number of unique bands was different for the two STPs profiles; no bands for *Pinus* × *rhaetica* and four bands for *Pinus uliginosa* (Table 1 and Figure 2). 

Importantly, these results are consistent with our previous findings, where the cytogenetic analysis demonstrated some differences in the DNA content and C-banding pattern of chromosome sets between *Pinus* × *rhaetica* and *P. uliginosa*, suggesting that the interchangeable use of their names as synonyms is unsupported [34]. Nevertheless, the unambiguous determination of their taxonomic status and phylogenetic origin requires further research, as it is clear that this challenging problem still remains unresolved. Recently, the same methodological approach, i.e., STP profiling and cytological analysis, was sufficient for the evaluation of the genetic diversity of species belonging to the *Solanaceae* Juss. family (*S. melongena* L., *S. xanthocarpum* L., *Datura alba* L., *Lycopersicon esculantum* L., and *Capsium annum* L.) [41].

While the densitometric analysis conducted here differentiates *Pinus* × *rhaetica* and *Pinus uliginosa* from each other, diagnostic bands for all three representatives of the *Pinus mugo* complex (Table 1) could be identified (Figure 2; in orange). The STP profiles of all three taxa from this complex represent two common bands with a relative mobility of Rf = 0.13 and Rf = 0.60. While the band Rf = 0.60 was also present in *Abies koreana*, it is still accurate to use it as a semi-diagnostic band for the *Pinus mugo* complex, as it is difficult to confuse *Abies* with *Pinus*, considering their morphology. Importantly, a specific diagnostic band (Rf = 0.40) of the entire *Pinus* section was identified, which was not present in either *Abies koreana*, *Pseudotsuga menziesii,* or *Pinus strobus* from the *Strobus* section. Corresponding observations on the existence of shared diagnostic bands in STPs profiles of closely related pines (*P. sylvestris*, *Pinus mugo,* and *Pinus uncinata*) from amongst twelve analyzed taxa were done by Schirone et al. [42]. The current analysis enabled the identification of diagnostic bands for the *Pinus mugo* complex and for the section *Pinus*.

### 2.2. Genetic Distance and Cluster Analysis

The obtained STPs profiles (Figure 1 and Figure 2) were transformed into a binary matrix to enable the calculation of Nei‘s genetic distance (GD) and the Jaccard similarity coefficient (JS) values, given in Table 2. The average value of the genetic distance was 0.506, which ranged from 0.238 for the pair *Pinus sylvestris* and *Pinus* × *rhaetica,* to 0.693 for the pair *Pinus nigra/Pinus strobus*. The genetic distance between *Pinus* × *rhaetica* and *Pinus uliginosa* equaled 0.361, while the two taxa were less distant from *Pinus mugo* (GD = 0.318), thus being their putative parent. As discussed earlier, *Pinus* × *rhaetica* was found to be more closely related to *Pinus sylvestris* than to *Pinus mugo*. It is noteworthy that the calculated genetic distance values were the largest among different taxa inside the genus *Pinus*, rather than between the taxa originating from different genera.

The Jaccard similarity coefficient oscillated between 6.7 and 51.7%, with an average for the eight taxa of 23.7%. The results showed that the largest similarity existed between *Pinus sylvestris* and *Pinus* × *rhaetica* (51.7%), and between *P. uliginosa* and *P. mugo* (47.1%); thus proving them to be the putative parent and the more similar descendant, respectively. Expectedly, the lowest Jaccard similarity coefficient was observed for the pair *Abies koreana* and *Pinus nigra*.

**Figure 3:**
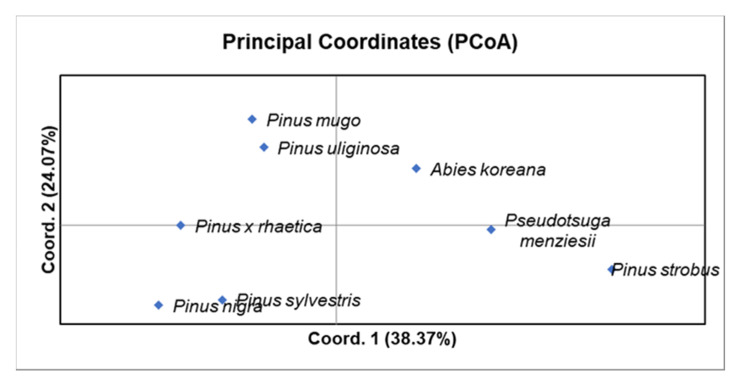
Principle coordinates analysis (PCoA) using Nei’s genetic distance (GD) for the eight taxa from the Pinaceae family.

Based on the principal coordinates analysis (PCoA; Figure 3), *Pinus uliginosa* is phylogenetically closer to *Pinus mugo* than to *Pinus sylvestris*, and *Pinus* × *rhaetica* is closer to *Pinus sylvestris* than to *Pinus mugo*. These results are consistent with the data derived directly from the analysis of the number of bands (Table 1), the Nei’s genetic distance, or the Jaccard similarity coefficient (Table 2). Overall, the two first coordinates of the PCA explained 62.44% of the observed variation. 

**Figure 4:**
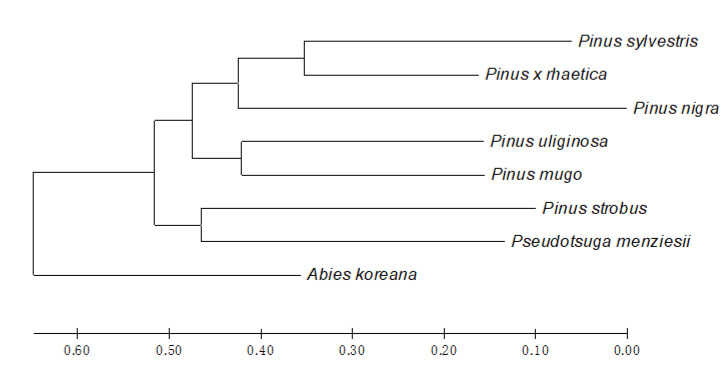
Evolutionary relationships of the Pinaceae taxa under study. The evolutionary history was inferred using the Neighbor-Joining method (NJ), based on the Jaccard distance (JD). The optimal tree with the sum of the branch length = 2.82700000 is shown. The tree is drawn to scale, with the branch lengths in the same units as those of the evolutionary distances used to infer the phylogenetic tree. Evolutionary analyses were conducted in MEGA X [43].

Finally, based on the Jaccard distance (JD), cluster analysis of the taxa was conducted and represented by the Neighbor Joining (NJ) dendrogram (Figure 4). The STP profiling divided the eight Pinaceae taxa into two main clusters. Cluster I is further divided into the following two subclusters: (i) comprising *Pinus mugo* and *Pinus uliginosa*, (ii) where *Pinus* × *rhaetica* is grouped with *Pinus sylvestris* and *Pinus nigra*. Cluster II groups two species, i.e., *Pinus strobus* and *Pseudotsuga menziesii*, while *Abies koreana* represent an outgroup. 

The evolutionary relationships revealed by the STP profiling conducted here are generally consistent with the commonly accepted taxonomic division of the Pinaceae family, with one exception, namely the position *Pinus strobus*. Based on the current data, *Pinus strobus* is subgrouped with *Pseudotsuga menziesii*, while it is typically grouped with the other taxa of the *Pinus* genus. Still, its distinct character is commonly known, and was the rationale behind dissecting separate sections: *Pinus* and *Strobus* inside the *Pinus* genus. 

The topology of the phylogenetic NJ tree provides interesting results regarding the position of *Pinus* × *rhaetica* and its relationship with the other pines, including postulated parental species. As shown in Figure 4, *Pinus* × *rhaetica* is grouped with *Pinus sylvestris*, and not with the remaining representatives of the *Pinus mugo* complex, i.e., *Pinus uliginosa* and *Pinus mugo*, as expected. Such grouping complies with the observations from the direct bands comparison (Table 1). In previous studies, the DNA sequence analysis of almost 6000 nt showed no differences between the taxa from the *Pinus mugo* complex [1]. However, in this study, the STP profiling showed that these closely related taxa differ, and, according to the tree-building method, they represent separate taxa. The corresponding conclusions were withdrawn from our previous chemotaxonomy studies, comprising essential oils and volatiles profiling in the pine species [35,36]. The qualitative and quantitative differences between the taxa in terms of essential oils and volatiles composition were high enough to propose a special chemotaxonomic key for the discrimination of *Pinus uliginosa*, *Pinus mugo,* and *Pinus uncinata* from the *Pinus mugo* complex

## 3. Materials and Methods

### 3.1. Plant Material

**Figure 5:**
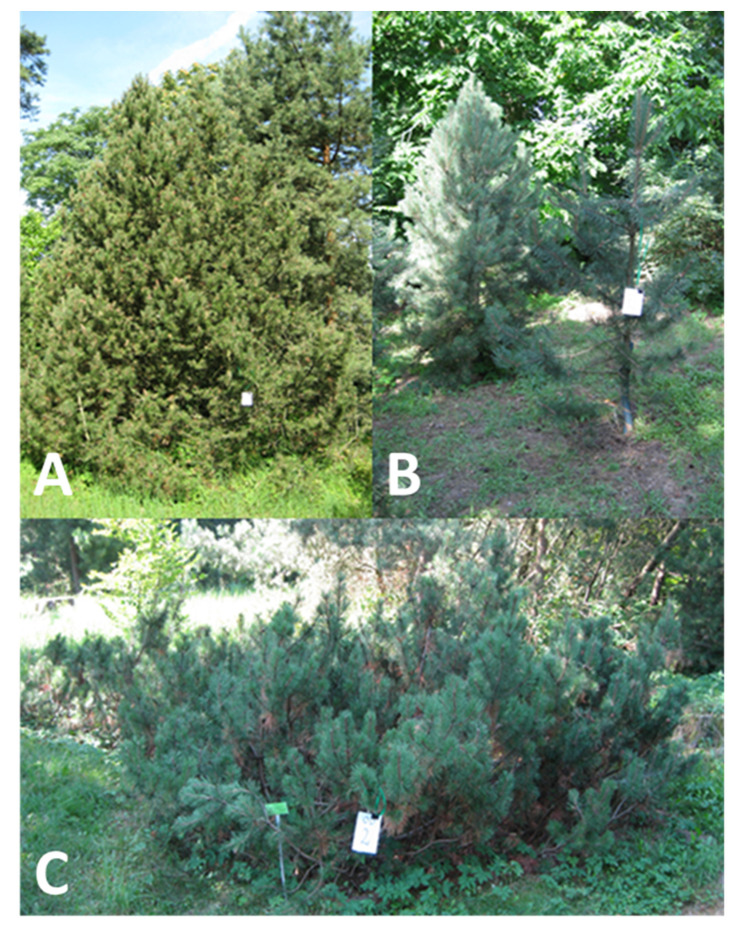
Morphology of the representatives of the *Pinus mugo* complex analyzed in this study. (**A**): *Pinus* × *rhaetica*, (**B**): *Pinus uliginosa*, (**C**): *Pinus mugo.*

The Pinaceae taxa investigated in the present study are listed in Table 3. The experiments were conducted using six representatives of the *Pinoideae* subfamily and a single representative from each *Abietoideae* and *Laricoideae* families. Figure 5 presents the *Pinus mugo*, *Pinus uliginosa,* and *Pinus* × *rhaetica* morphology. The seeds for the studies were kindly provided by the Botanical Garden in Lodz (BGL) and by the Dendrological Garden, Poznan University of Life Sciences (DGP), both located in Poland.

### 3.2. Seed Total Protein (STP) Extraction

The STPs were extracted from air-dried seeds without coats using 0.05 M Tris-HCl buffer (pH 8.0) and 2% 2-mercaptoethanol, according to the procedure by Tomooka [44]. After thorough vortexing, the homogenate was shaken for 1 h at 150 rpm at room temperature, followed by centrifugation at 12,000 rpm for 5 min at 4 °C. The total protein concentration in the supernatant was measured according to Popov et al. [45], using the precipitation method with an acidic, methanolic amido black 10B solution. The extracted STPs were kept at −20 °C until further use.

### 3.3. SDS-PAGE Electrophoresis

The STP extracts were separated in 14% polyacrylamide (PAA) gel under denaturating conditions (SDS-PAGE—sodium dodecyl sulfate polyacrylamide gel electrophoresis), according to the Laemmli [46] method. Eighty µg of the STP extracts were loaded on the gel. Unstained protein molecular weight marker (14.4 kDa to 116 kDa; Thermo Fisher Scientific, Inc.) was used as an electrophoretic mobility marker. Electrophoresis was conducted under 90V for approximately 6 h in Hoefer™ SE 600 Chroma Vertical Electrophoresis System (Thermo Fisher Scientific, Inc.). The gels were stained with Coomassie Brilliant Blue R-250 (Bio-Rad), according to Hames and Rickwood [47]. Image acquisition was done using The Quantum documentation imaging system (Vilber). All of the analyses were conducted in biological triplicate. Computational analysis was conducted using bands that were unambiguously identified and clearly separated in all of the replicates. 

### 3.4. Band Scoring and Data Analysis

GelAnalyzer 19.1 [48] software was used to analyze the acquired images of the SDS-PAGE gels, using the following settings: detect peaks on all of the lanes (peak threshold: 1; peak min. height: 10; peak max. width in % on lane profile length: 5%) and detect background on all lines option (rolling ball and peak width tolerance in % of lane profile length: 10%). The STP profiles were transformed into a binary system of 0 (absence) and 1 (presence), according to their relative mobility value (Rf) [42]. The obtained binary matrix was used to calculate the Nei genetic distance (GD) and Jaccard similarity coefficient (JS). The Jaccard distance (JD) was calculated using the following formula: 1 − value of the Jaccard similarity coefficient. Cluster analysis was also performed using the Neighbor Joining Method (NJ) and Jaccard distance, as well as principal coordinates analysis (PCoA) based on Nei‘s genetic distance. All of the analyzes and calculations were carried out using the following programs: GenAIEx 6.501 [49,50], MVSP 3.22 [51], and MEGA X [43].

## 4. Conclusions

In this study, we used the STP patterns to search for taxonomically interesting differences among three closely-related pine taxa from the *Pinus mugo* complex and five more distant species from the Pinaceae family. Based on the obtained results, it can be concluded that the STP profiling revealed diagnostic electrophoretic patterns for all of the analyzed taxa from the Pinaceae family, and also allowed for discriminating closely related pine taxa, even those indistinguishable by chloroplast DNA barcodes. The results of the STPs patterns obtained here corroborate our previous findings conducted using the chemotaxonomic approach. It would be interesting to challenge this methodology with the other taxonomic puzzles occurring in the other plants groups.

## Figures and Tables

**Table 1:** Summary of STP profiles for the Pinaceae taxa under study. Unique bands for taxa are given in bold. The single band common to all of the taxa is given in italics. N_B_—number of bands; N_UB_—number of unique bands.

No.	Taxon	N_B_	Rf Range	Bands Relative Mobility Value (Rf)	N_UB_
1	*A. koreana*	13	0.09–0.72	0.09, 0.11, 0.15, 0.18, *0.22*, 0.24, 0.30, 0.41, **0.48**, 0.50, 0.60, **0.70**, 0.72	2
2	*P. nigra*	21	0.05–0.85	0.05, 0.07, **0.10**, 0.12, 0.15, 0.17, 0.19, 0.20, *0.22*, 0.28. 0.32. 0.33, 0.40, **0.45**, 0.54, **0.56**, 0.58, 0.71, **0.75**, 0.81, **0.85**	5
3	*P. strobus*	24	0.07–0.87	0.07, 0.09, 0.12, 0.14, 0.15, *0.22*, 0.25, 0.29, 0.32, 0.33, **0.35**, 0.39, **0.44**, 0.46, 0.50, 0.55, 0.59, **0.61**, 0.63, **0.67**, **0.76**, **0.78**, 0.82, **0.87**	7
4	*P. menziesii*	19	0.09–0.82	0.09, 0.12, 0.14, 0.15, 0.16, 0.18, *0.22*, 0.23, 0.29, 0.33, 0.39, 0.41, 0.46, **0.57**, **0.65**, **0.68**, 0.72, 0.79, 0.82	3
5	*P. sylvestris*	20	0.06–0.81	0.06, 0.09, 0.11, 0.14, 0.17, 0.19, 0.20, *0.22*, 0.23, 0.27, **0.31**, 0.33, 0.40, 0.46, 0.54, 0.59, **0.64**, 0.71, 0.79, 0.81	2
6	*P.* × *rhaetica*	24	0.06–0.82	0.06, 0.09, 0.11, 0.12, 0.13, 0.14, 0.16, 0.17, 0.19, 0.20, *0.22*, 0.23, 0.28, 0.30, 0.32, 0.33, 0.40, 0.46, 0.54, 0.58, 0.60, 0.71, 0.79, 0.82	0
7	*P. uliginosa*	28	0.04–0.86	**0.04**, 0.06, 0.09, 0.12, 0.13, 0.15, 0.16, 0.17, 0.19, 0.20, *0.22*, 0.24, 0.25, 0.27, 0.32, 0.33, 0.40, 0.46, 0.55, 0.58, 0.60, 0.63, **0.69**, 0.72, **0.77**, 0.80, 0.82, **0.86**	4
8	*P. mugo*	22	0.05–0.82	0.05, 0.09, 0.11, 0.13, 0.15, 0.16, 0.17, 0.19, *0.22*, 0.24, 0.28, 0.30, 0.32, **0.34**, 0.40, 0.46, 0.55, 0.60, 0.72, **0.74**, 0.80, 0.82	2

**Table 2:** Nei’s genetic distance (GD; below diagonal) and Jaccard similarity coefficient (JS; above diagonal) based on the STP profiles analysis.

	*A. koreana*	*P. nigra*	*P. strobus*	*P. menziesii*	*P. sylvestris*	*P.* × *rhaetica*	*P. uliginosa*	*P. mugo*
*A. koreana*	-	0.067	0.121	0.231	0.100	0.156	0.171	0.296
*P. nigra*	0.606	-	0.103	0.086	0.258	0.265	0.205	0.171
*P. strobus*	0.579	0.693	-	0.303	0.158	0.200	0.268	0.179
*P. menziesii*	0.361	0.663	0.428	-	0.219	0.303	0.237	0.206
*P. sylvestris*	0.526	0.428	0.663	0.476	-	0.517	0.263	0.200
*P.* × *rhaetica*	0.526	0.383	0.663	0.428	0.238	-	0.444	0.438
*P. uliginosa*	0.579	0.579	0.606	0.579	0.552	0.361	-	0.471
*P. mugo*	0.340	0.526	0.663	0.526	0.552	0.318	0.318	-

**Table 3:** The Pinaceae taxa investigated in the present study. The seeds origin: BGL—The Botanical Garden in Lodz (Poland); DGP—The Dendrological Garden, Poznan University of Life Sciences (Poland). * taxa belonging to the *Pinus mugo* complex.

No.	Taxon	Subfamily	Genus	Subgenus/Section	Seeds Origin
1	*Abies koreana* E.H. Wilson	*Abietoideae*	Abies	-	BGL
2	*Pinus nigra* Arn.	*Pinoideae*	Pinus	Pinus	BGL
3	*Pinus strobus* L.	*Pinoideae*	Pinus	Strobus	BGL
4	*Pseudotsuga menziesii* (Mirbel) Franco	*Laricoideae*	Pseudotsuga	-	BGL
5	*Pinus sylvestris* L.	*Pinoideae*	Pinus	Pinus	BGL
6	*Pinus* × *rhaetica* Brügger*	*Pinoideae*	Pinus	Pinus	DGP
7	*Pinus uliginosa* Neumann ex Wimmer*	*Pinoideae*	Pinus	Pinus	DGP
8	*Pinus mugo* Turra*	*Pinoideae*	Pinus	Pinus	DGP
